# Successful esophageal endoscopic submucosal dissection with a transoral endoscope after stepwise scope bougienage of post‐endoscopic submucosal dissection stricture

**DOI:** 10.1002/jgh3.12437

**Published:** 2020-10-22

**Authors:** Maiko Takita, Ken Ohata, Ryoju Negishi, Yohei Minato, Takashi Muramoto

**Affiliations:** ^1^ Department of Gastrointestinal Endoscopy NTT Medical Center Tokyo Tokyo Japan

**Keywords:** endoscopic resection, esophageal cancer, post‐endoscopic submucosal dissection stricture, scope bougienage

## Abstract

Endoscopic submucosal dissection (ESD) for extensive esophageal cancer inevitably causes a post‐ESD stricture. It may be difficult to perform additional ESD if a new lesion develops on the anus side of the post‐ESD stricture. We sometimes perform balloon dilation of post‐ESD stricture in advance, so we could perform ESD using a transoral scope; however, there is a risk of balloon dilation causing severe tearing of the lesions if it is located near the stricture. A 68‐year‐old man who had undergone ESD for esophageal cancer several times was diagnosed with early esophageal cancer. The lesion was located near the anus side of the post‐ESD stricture. Unfortunately, the lesion was located on another post‐ESD scar. Although ESD using a transnasal scope was a useful option, it was expected to be challenging as the submucosal layer was thought to have severe fibrosis. We attempted to perform ESD with a transoral endoscope after stepwise scope bougienage of post‐ESD stricture.

## Graphical abstract

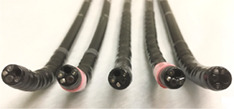

Endoscopic submucosal dissection (ESD) for extensive esophageal cancer inevitably causes a post‐ESD stricture. It may be difficult to perform additional ESD if a new lesion develops on the anus side of the post‐ESD stricture. We attempted to perform ESD with a transoral endoscope after stepwise scope bougienage of the post‐ESD stricture.

## Introduction

Esophageal strictures are common after endoscopic submucosal dissection (ESD) of extensive tumors. Patients who have undergone ESD for such lesions often develop a post‐ESD stricture that a transoral scope cannot pass through. As metachronous esophageal cancer occurs frequently,^1^ it may be difficult to perform additional ESD if a new lesion develops on the anus side of the post‐ESD stricture. We sometimes performed balloon dilation of post‐ESD stricture in advance, so we could perform ESD using transoral scope; however, there is a risk of balloon dilation causing severe tearing of the lesion if it is located near the anus side of the stricture. Here, we report a case of successful esophageal ESD with a transoral endoscope after stepwise scope bougienage of post‐ESD stricture.

## Case report

**Figure 1:**
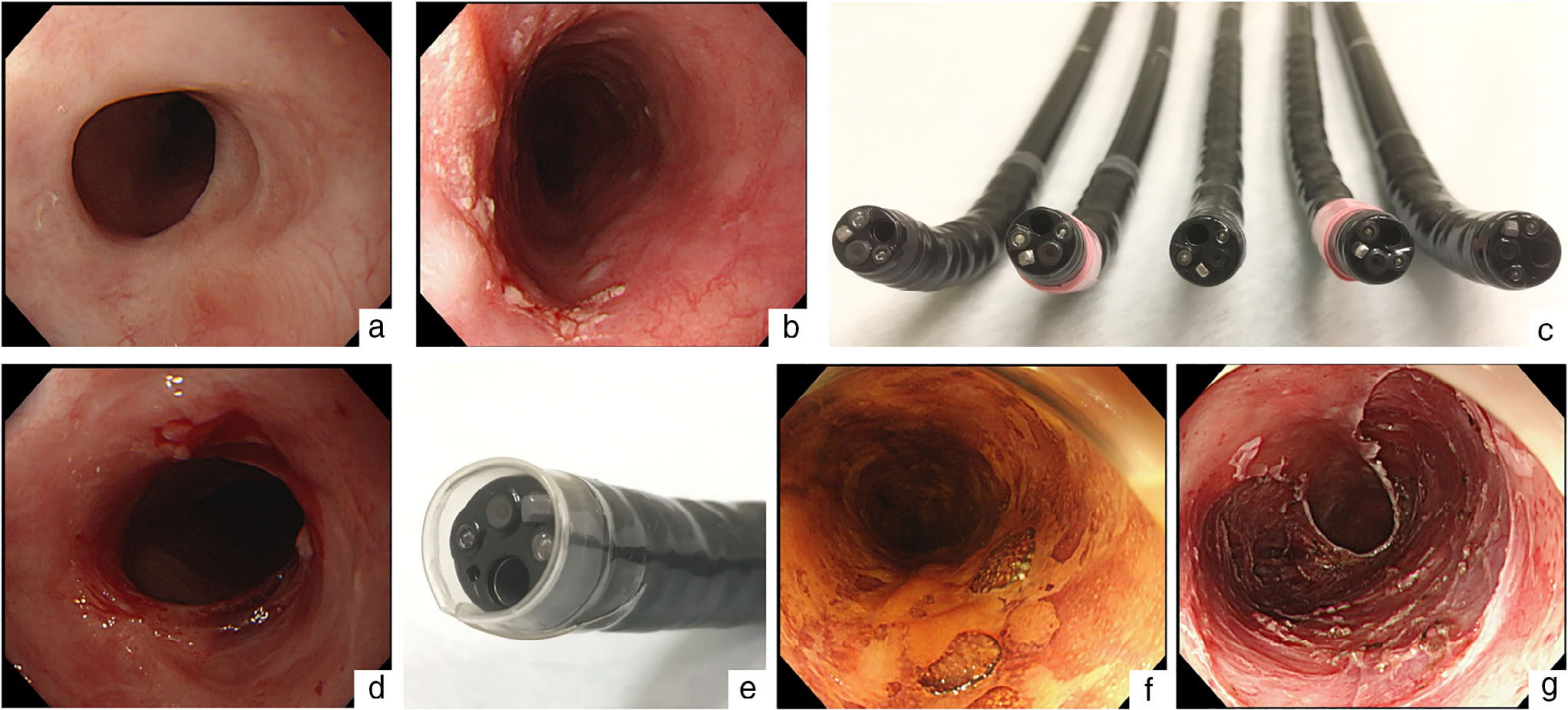
Endoscopic submucosal dissection (ESD) for early esophageal cancer developed on the anus side of the post‐ESD stricture after stepwise scope bougienage. (a) A post‐ESD stricture in the middle thoracic esophagus. (b) The lesion is located near the anus side of the post‐ESD stricture. (c) Bougienage is performed by gradually increasing the outer diameter of the scope. GIF‐H290, GIF‐H290 wrapped in plastic tape; PCF‐PQL, PCF‐PQL wrapped in tape, and GIF‐H290T from the left. (d) Stricture after stepwise scope bougienage. (e) A handmade distal endoscope attachment made with plastic tape. (f) Accurate diagnosis of disease extension is possible without damaging the lesion by bougienage. (g) Mucosal defect after ESD.

A 68‐year‐old man who had undergone ESD for esophageal cancer several times was diagnosed with early esophageal cancer. The lesion was a superficially depressed lesion that occupied approximately half of the circumference and was located near the anus side of the post‐ESD stricture (Fig. 1a,b). Unfortunately, the lesion was located on another post‐ESD scar. Because the submucosal layer was thought to have severe fibrosis, ESD using a transnasal scope was expected to be extremely difficult. Although the stricture had a diameter that the GIF‐H290 (diameter: 8.9 mm; Olympus, Tokyo, Japan) could barely pass through, it is not suitable for endoscopic treatment as it has poor suction capacity and no water supply function. In addition, the lumen had to be dilated to the extent that the scope with the distal attachment could be moved without resistance. Therefore, we attempted to perform ESD with the GIF‐H290T (diameter: 9.9 mm; Olympus), which is commonly used for endoscopic treatment after bougienage. Because balloon dilation carried the risk of tearing the lesion due to proximity to the stricture, we attempted to perform stepwise scope bougienage (Fig. 1c). First, we wrapped plastic tape around the GIF‐H290 to increase its diameter to a size that could pass through the stricture. Then, we used PCF‐PQL (diameter: 9.2 mm: Olympus) and wrapped it with tape. The diameter of the endoscope was increased by about 2–3 mm after winding the tape four times. We then performed bougienage with the GIF‐H290T (diameter: 9.9 mm). Finally, the stricture was extended so that the GIF‐H290T, with a handmade distal endoscope attachment with plastic tape, could pass through (Fig. 1d).^2^ Although it was difficult to pass a commercially available attachment through the stricture due to its thickness, the handmade attachment was sufficient to pass the stricture (Fig. 1e). The lesion was successfully endoscopically resected without any complications despite severe fibrosis (Fig. 1f,g). The pathological examination showed a squamous cell carcinoma, with pT1a‐LPM without lymphovascular infiltration. Six months after ESD, the transoral scope could pass through without difficulty, and the patient remained free of dysphagia.

## Discussion

ESD for extensive esophageal cancer inevitably causes a post‐ESD stricture. It has been reported that a mucosal defect involving more than three‐quarters of the esophageal circumference after endoscopic resection (ER) is a risk factor for post‐ESD stricture.^3^, ^4^, ^5^ Even if epithelialization is achieved without severe stricture, some patients still have some degree of post‐ESD stricture, and sometimes, the transoral scope cannot pass through. In these patients, if a new metachronous lesion develops on the anus side of the post‐ESD stricture, it is difficult to perform additional ESD. In such cases, we usually perform ESD after balloon dilation. Balloon dilation is useful; however, there is a possibility of perforation.^6^ In addition, because the balloon expands at once, it is likely to tear the lesions if it is located near the stricture. Recently, ESD performed using a transnasal scope for patients with esophageal strictures has been reported.^7^ Although this is a useful option, treatment with a transnasal scope is challenging due to poor visibility and maneuverability compared with a transoral scope. Moreover, they have poor suction capacity and no water supply function. For these reasons, it is difficult even for a fairly skilled endoscopist to perform ESD using a transnasal scope. Furthermore, if the lesion is on a post‐ESD scar, as in this case, treatment using a transnasal scope will be extremely difficult even for them. Fortunately, the stricture in this case had a diameter that the GIF‐H290 (diameter: 8.9 mm) could barely pass through, so we attempted to perform ESD with the GIF‐H290T (diameter: 9.9 mm), which is commonly used in esophageal ESD after stepwise scope bougienage. This is a method used to expand the esophagus by gradually increasing the diameter of the scope; we have previously reported the usefulness of this method for esophageal stricture after ESD.^8^ This procedure also has the risk of tearing esophageal mucosa, similar to balloon dilation. However, it can extend the lumen in units of mm by using plastic tape while assessing resistance by hand, and the diameter of the esophagus is increased gradually so that damage to the esophagus mucosa is often minimal compared to the balloon.

Another important aspect in this case is the use of the handmade distal endoscope attachment instead of the commercially available one. Needless to say, the distal endoscope attachment is essential for endoscopic treatment.^9^ However, in patients with an esophageal stricture, the distal attachment is often found to increase the diameter of the scope tip and prevents it from passing through the stricture. The handmade distal endoscope attachment used this time, made with plastic tape, only slightly increased the tip diameter of the scope, which is useful in such cases.

Interestingly, the mucosal defect subsequently became epithelialized without worsening of the stricture in this case. It should be noted that a new post‐ESD scar on the anus side led to traction of the stricture and a slight extension of the original lumen. As a result, the mild dysphagia the patient was feeling prior to treatment improved.

In conclusion, we report a case of successful esophageal ESD with an oral endoscope after stepwise scope bougienage of post‐ESD stricture. If a patient with a post‐ESD stricture develops metachronous esophageal cancer only on the anus side of the stricture, this method offers an alternative for such cases.

## References

[1] Katada C , Yokoyama T , Yano T *et al* Alcohol consumption and multiple dysplastic lesions increase risk of squamous cell carcinoma in the esophagus, head, and neck. Gastroenterology. 2016; 151: 860–9. 27492616 10.1053/j.gastro.2016.07.040

[2] Kurebayashi M , Sakai E , Suzuki Y , Ohata K . Usefulness of a handmade distal endoscope attachment with a transparent tape. VideoGIE. 2020; 5: 226–8. 32529152 10.1016/j.vgie.2020.02.012 PMC7280143

[3] Katada C , Muto M , Manabe T , Boku N , Ohtsu A , Yoshida S . Esophageal stenosis after endoscopic mucosal resection of superficial esophageal lesions. Gastrointest. Endosc. 2003; 57: 165–9. 12556777 10.1067/mge.2003.73

[4] Ono S , Fujishiro M , Niimi K *et al* Predictors of postoperative stricture after esophageal endoscopic submucosal dissection for superficial squamous cell neoplasms. Endoscopy. 2009; 41: 661–5. 19565442 10.1055/s-0029-1214867

[5] Shi Q , Ju H , Yao LQ *et al* Risk factors for postoperative stricture after endoscopic submucosal dissection for superficial esophageal carcinoma. Endoscopy. 2014; 46: 640–4. 24830402 10.1055/s-0034-1365648

[6] Yoda Y , Yano T , Kaneko K *et al* Endoscopic balloon dilation for benign fibrotic strictures after curative nonsurgical treatment for esophageal cancer. Surg. Endosc. 2012; 26: 2877–83. 22543993 10.1007/s00464-012-2273-9

[7] Nakamura M , Shiroeda H , Tahara T *et al* Endoscopic submucosal dissection of an esophageal tumor using a transnasal endoscope without sedation. Endoscopy. 2014; 46: E115–6. 24676817 10.1055/s-0034-1364885

[8] Takita M , Sakai E , Ohata K . Stepwise scope bougienage for esophageal stricture after endoscopic submucosal dissection for circumferential lesions. Dig. Endosc. 2019; 31: 719. 31411355 10.1111/den.13513

[9] Yamamoto H , Kawata H , Sunada K *et al* Successful en bloc resection of large superficial tumors in the stomach and colon using sodium hyaluronate and small caliber tip transparent hood. Endoscopy. 2003; 35: 690–4. 12929067 10.1055/s-2003-41516

